# Supplementary material for: Identification of Freezing-Responsive microRNAs and Their Targets in Chinese Jujube by Small RNA and Degradome Sequencing
Source: Int J Mol Sci. 2026 Sep 4;27(17):7912. doi: 10.3390/ijms27177912 (PMC13566890; doi:10.3390/ijms27177912)
Supplement: Supplementary file 1 [file ijms-27-07912-s001.zip › Supplementary Material S2/novel_miR_186.pdf]

Provisional ID	:	novel_miR_186
Score total	:	0.8
Score for star read(s)	:	-1.3
Score for read counts	:	0
Score for mfe	:	0.5
Score for randfold	:	1.6
Score for cons. seed	:	
Total read count	:	5009
Mature read count	:	2845
Loop read count	:	27
Star read count	:	2137

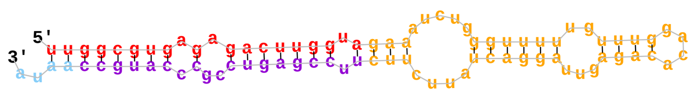

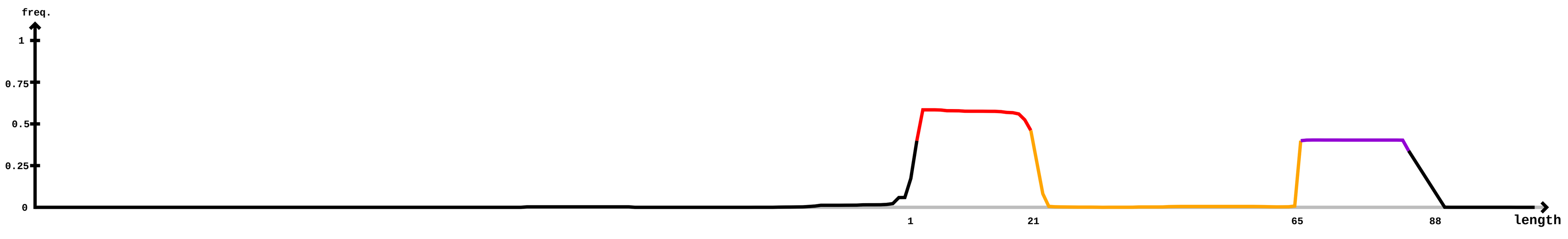

Mature / Star

```
5'- ucacgugauaacgcguccuuugcuuuuuauuuuuuauuugaaauucaaaaaaauaauaauaaagauauauuuauauaauuguggaugugaaagaagaacuacuaccacuacacagagugauuuuuuuaugaggcugagaaaauuguuggcgugagagacuugguagaaaucuggguuuuuguuuggacacagaguuaggacuauucuucuuccgaguccgcccaugccaauaauuuccuccgucuuuuc -3'  obs
    ucacgugauaacgcguccuuugcuuuuuauuuuuuauuugaaauucaaaaaaauaauaauaaagauauauuuauauaauuguggaugugaaagaagaacuacuaccacuacacagagugauuuuuuuaugaggcugagaaaauuguuggcgugagagacuugguagaaaucuggguuuuuguuuggacacagaguuaggacuauucuucuuccgaguccgcccaugccaauaauuuccuccgucuuuuc       exp
    ..(((((....)))))......((((((((.(((.(((((...))))).))).))))).)))).((((((((((....))))))))))((((((..(((.((.........)))))..))))))...(((((.(((.((((((((((((((.(.(((((((.((((.....((((((..((((....))))...))))))....)))).)))))))..).)))))))))))))).))).)))))....
```

read	reads	mm	sample
guggaugugaaagaagaa	6	0	JT-2
guggaugugaaagaagaa	9	0	JT-3
gauuuuuuuaugaggcugagaa	1	0	LT-1
uuuuuaugaggcugagaaaau	1	0	JCK-2
uuuuuaugaggcugagaaaau	1	0	JCK-1
uuuuuaugaggcugagaaaauug	3	0	JCK-2
uuuuaugaggcugagaaaauuguuggcgug	2	0	LT-1
uuuaugaggcugagaaaauuguuggcgug	1	0	JT-1
uuaugaggcugagaaaauug	1	0	LT-1
uuaugaggcugagaaaauuguuggcgu	1	0	LT-2
uuaugaggcugagaaaauuguuggcgu	1	0	LT-1
uaugaggcugagaaaauuguuggcgug	2	0	LT-1
augaggcugagaaaauuguuggU	1	1	LT-2
augaggcugagaaaauuguuggc	1	0	LT-1
augaggcugagaaaauuguuggc	2	0	LT-3
augaggcugagaaaauuguuggcg	3	0	JT-3
augaggcugagaaaauuguuggcg	5	0	JT-2
ugaggcugagaaaauuguugg	3	0	LCK-3
ugaggcugagaaaauuguugg	6	0	LT-2
ugaggcugagaaaauuAuugg	1	1	JCK-3
ugaggcugagaaaauuguuggc	1	0	JCK-3
ugaggcugagaaaauuguuggc	1	0	JCK-2
ugaggcugagaaaauuguuggcgug	1	0	JCK-1
ugaggcugagaaaauuguuggcgugaga	1	0	LT-1
gaggcugagaaaauuguuggc	1	0	LT-2
gaggcugagaaaauuguuggc	17	0	LCK-3
gaggcugagaaaauuguuggcg	2	0	LT-3
gaggcugagaaaauuAuuggcgug	2	1	JCK-3
gaggcugagaaaauuguuggcgug	1	0	JCK-1
gaggcugagaaaauuAuuggcgug	1	1	JCK-2
gaggcugagaaaauuguuggcgug	2	0	LT-3
cugagaaaauuguuggcgug	1	0	LT-3
cugagaaaauuguuggcguga	1	0	LT-3

Mature Star

ucacgugauaacgcguccuuugcuuuuuauuuuuuauuugaaauucaaaaaaauaauaauaaagauauauuuauauaauuguggaugugaaagaagaacuacuaccacuacacagagugauuuuuuuaugaggcugagaaaauuguuggcgugagagacuugguagaaaucuggguuuuuguuuggacacagaguuaggacuauucuucuuccgaguccgcccaugccaauaauuuccuccgucuuuuuc

ugagaaaauuguuggcgugagagacu	1	0	LT-3
gagaaaauugAuggcgugagaga	1	1	LT-3
agaaaauuguuggcgugagag	2	0	LT-1
agaaaauuguuggcgugagag	3	0	LT-3
agaaaauuguuggcgugagagacu	6	0	LCK-3
gaaaauuAuuggcgugagagacuuggua	1	1	JCK-1
aaauuAuuggcgugagagacuugg	1	1	JCK-1
aauuguuggcgugagagac	6	0	LCK-3
aauuguuggcgugagagac	1	0	LT-1
aCuuguuggcgugagagacu	1	1	LT-3
aauuguuggcgugagagacuugg	2	0	JCK-3
aauuguuggcgugagagacuugg	1	0	JCK-2
auuguuggcgugagagac	2	0	LT-2
auuAuuggcgugagagacu	1	1	JCK-2
auuguuggcgugagagacuug	1	0	LT-3
auuguuggcgugagagacuug	2	0	LT-1
auuguuggcgugagagacuuggua	1	0	JCK-1
auuAuuggcgugagagacuuggua	1	1	JCK-1
auuguuggcgugagagacuuggua	4	0	LT-2
auuguuggcgugagagacuuggua	4	0	LCK-3
auuguuggcgugagagacuuggua	3	0	LT-1
auuAuuggcgugagagacuuggua	2	1	JCK-2
auuAuuggcgugagagacuuggua	2	1	JCK-3
auuguuggcgugagagacuuggua	4	0	LT-3
uuAuuggcgugagagacu	1	1	JCK-3
uuguuggcgugagagacu	2	0	LT-1
uuAuuggcgugagagacu	3	1	JCK-2
uuguuggcgugagagacu	2	0	JCK-2
uuguuggcgugagagacu	3	0	LT-3
uuguuggcgugagagacu	5	0	LCK-3
uuguuggcAugagagacu	1	1	LCK-3
uuguuggcgugagagacuu	2	0	LT-1
uuAuuggcgugagagacuu	1	1	JCK-2
uuAuuggcgugagagacuu	3	1	JCK-3
uuguuggcgugagagacuu	1	0	JCK-1
uuguuggcgugagagacuug	8	0	LCK-3
uuguuggcgugagagacuug	2	0	LT-2
uuAuuggcgugagagacuug	1	1	JCK-2
uuAuuggcgugagagacuugg	41	1	JCK-2
uuguuggcgugagagacuugg	7	0	LCK-3
uuguuggcgugagagacuugg	10	0	LT-2
uuguuggcgugagagacuugg	5	0	LT-1
uuAuuggcgugagagacuugg	11	1	JT-1
uuAuuggcgugagagacuugg	32	1	JCK-1
uuCuuggcgugagagacuugg	1	1	JCK-2
uuguuggcgugagagacuugg	1	0	JCK-3
uuguuggcgugagagacuugg	6	0	LT-3
uuAuuggcgugagagacuugg	29	1	JCK-3
uuguuggcgugagagacuuggu	1	0	JCK-3
uuguuggcgugagagacuuggu	1	0	JCK-2
uuAuuggcgugagagacuuggu	1	1	JCK-1
uuguuggcgugagagacuuggu	1	0	JCK-1
uuguuggcgugagagacuuggu	1	0	LT-1
uuguuggcgugagagacuuggu	1	0	LT-2
uuguuggcgugagagacuuggua	2	0	LT-3
uuAuuggcgugagagacuuggua	4	1	JCK-3
uuAuuggcgugagagacuuggua	1	1	JCK-1
uuAuuggcgugagagacuuggua	3	1	JCK-2
uuguuggcgugagagacuuggua	1	0	LT-1
uguuggcgugagagacuu	1	0	LT-1
uguuggcgugagagacuugg	1	0	LT-1
uguuggcgugagagacuuggua	2	0	LT-3
Auuggcgugagagacuug	6	1	JCK-1
Auuggcgugagagacuug	7	1	JCK-3
Auuggcgugagagacuug	12	1	JCK-2
Auuggcgugagagacuug	2	1	JT-1
Auuggcgugagagacuugg	3	1	JCK-3
Auuggcgugagagacuugg	4	1	JCK-2
Auuggcgugagagacuugg	3	1	JCK-1
Auuggcgugagagacuugg	7	1	JT-2

Mature Star

ucacgugauaacgcguccuuugcuuuuuauuuuuuauuugaaauucaaaaaaauaauaauaaagauauauuuauauaauuguggaugugaaagaagaacuacuaccacuacacagagugauuuuuuuaugaggcugagaaaauuguuggcgugagagacuugguagaaaucuggguuuuuguuuggacacagaguuaggacuauucuucuuccgaguccgcccaugccaauaauuuccuccgucuuuuuc

Auuggcgugagagacuugg	5	1	JT-3
Auuggcgugagagacuuggu	3	1	JT-2
Auuggcgugagagacuuggu	55	1	JCK-2
Cuuggcgugagagacuuggu	1	1	JCK-2
Auuggcgugagagacuuggu	24	1	JCK-3
Auuggcgugagagacuuggu	3	1	JT-3
Auuggcgugagagacuuggu	14	1	JCK-1
Auuggcgugagagacuuggua	9	1	JT-1
guuggcgugagagacuuggua	2	0	LT-1
Auuggcgugagagacuuggua	144	1	JCK-3
Cuuggcgugagagacuuggua	2	1	JCK-3
Auuggcgugagagacuuggua	81	1	JCK-1
Auuggcgugagagacuuggua	171	1	JCK-2
Cuuggcgugagagacuuggua	2	1	JCK-2
Auuggcgugagagacuugguag	20	1	JCK-1
Auuggcgugagagacuugguag	17	1	JCK-2
Auuggcgugagagacuugguag	18	1	JCK-3
guuggcgugagagacuugguagaa	1	0	LT-3
uuggcgugagagacuugg	3	0	LT-3
uuggcgugagagacuugg	4	0	LT-2
uuggcgugagagacuugg	2	0	LT-1
uuggcgugagagacuugg	4	0	JCK-3
uuggcgugagagacuugg	6	0	JCK-2
uuggcgugagagacuugg	2	0	LCK-3
uNggcgugagagacuuggu	1	1	JCK-2
uuggcgugagagacuuggu	2	0	JT-2
uuggcgugagagacuuggu	11	0	JT-1
uuggcgugagagacuuggu	15	0	LT-1
uuggcgugagagacuuggu	7	0	LT-3
uuggcgugagagacuuggu	20	0	LCK-1
uuggcgugagagacuuggu	24	0	LCK-2
uuggcgugagagacuuggu	1	0	JT-3
uuggcgugagagacuuggu	17	0	LT-2
uuggcgugagagacuugUu	1	1	JCK-3
uuggcgugagagacuuggu	23	0	JCK-3
uuggcgugagagacuuggu	41	0	JCK-2
uuggcgugagagacuuggu	3	0	LCK-3
uuggcgugagagacuuggu	18	0	JCK-1
uuggcgugagagaAuuggu	1	1	JT-2
uuggcgugagagacuugguC	1	1	LT-1
uuggcgugagCgacuuggua	2	1	JT-1
uuggcgugagagacuugguU	10	1	JCK-3
uuggcgugagagacuuggua	6	0	JT-3
uuggcgugagagaAuuggua	1	1	JCK-3
uuggcgugagagacuuAgua	1	1	JCK-2
uuggcgugagagacuuggua	41	0	LCK-3
uuggcgugagagacuugguC	2	1	LT-3
uuggcgugagagaAuuggua	2	1	JCK-1
uuggcgugagagacuuggua	154	0	JCK-1
uuggcgugagagacuugguC	1	1	JCK-2
uuggcgugagagacuuggua	32	0	JT-1
uuggcgugagCgacuuggua	1	1	JCK-2
uuggcgugagagacuugguU	4	1	JCK-2
uuggcgugagagacuuggua	13	0	LT-1
uuggcgugagaAacuuggua	1	1	JCK-2
uuggcgugagagacCuggua	1	1	JCK-3
uuggcgugagaUacuuggua	1	1	LT-2
uuggcgugagagacuugguU	4	1	LCK-3
uuggcgugagagacuugguU	3	1	JCK-1
uuggcgugagagacuuggua	183	0	JCK-3
uuggcgugagagacuuggua	21	0	LT-2
uuggcgugCgagacuuggua	1	1	JT-1
uuggcgugagagacuuggua	7	0	JT-2
uuggcgugagagacuugCua	1	1	JCK-1
uuggcgugagagacuugguU	2	1	LT-3
uuggcgugagagacuuggua	46	0	LT-3
uuggcgugagagacuuggua	208	0	JCK-2
uuggcgugagagacuugguag	7	0	LT-3
uuggcgugagagacuugguag	13	0	LT-2
uuggcgugagCgacuugguag	1	1	JCK-3

Mature **Star**

```
ucacgugauaacgcguccuuugcuuuuuauuuuuuauuugaaauucaaaaaaauaauaauaaagauauauuuauauaauuguggaugugaaagaagaacuacuaccacuacacagagugauuuuuuuaugaggcugagaaaauuguuggcgugagagacuugguagaaaucuggguuuuuguuuggacacagaguuaggacuauucuucuuccgaguccgcccaugccaauaauuuccuccgucuuuuc
.................................................................................................................................................uuggcgugagagacuugguag...................................................................................   62   0   JCK-2
.................................................................................................................................................uuggcgugagagacuugguaA...................................................................................   3   1   JCK-3
.................................................................................................................................................uuggcgugagagacuugguaA...................................................................................   2   1   JCK-1
.................................................................................................................................................uuggcgugagaNacuugguag...................................................................................   1   1   JCK-1
.................................................................................................................................................uuggcgugagagacuugguag...................................................................................   40   0   JT-1
.................................................................................................................................................uuggcgugagagacuugguag...................................................................................   8   0   LT-1
.................................................................................................................................................uuggcgugagagacuugguag...................................................................................   27   0   JCK-3
.................................................................................................................................................uuggcgugagagacuugguag...................................................................................   31   0   JCK-1
.................................................................................................................................................uuggcgugagagacuugguag...................................................................................   2   0   LCK-3
.................................................................................................................................................uuggcgugagagacuugguaA...................................................................................   1   1   JCK-2
.................................................................................................................................................uuggcgugagagacuugguagC..................................................................................   1   1   JT-2
.................................................................................................................................................uuggcgugagagacuugguagC..................................................................................   5   1   JT-3
.................................................................................................................................................uuggcgugagagacuugguagC..................................................................................   1   1   JCK-1
.................................................................................................................................................uuggcgugagagacuugguagU..................................................................................   14   1   JCK-3
.................................................................................................................................................uuggcgugagagacuugguagU..................................................................................   3   1   LT-1
.................................................................................................................................................uuggcgugagagacuugguagU..................................................................................   18   1   JCK-1
.................................................................................................................................................uuggcgugagagacuugguagC..................................................................................   1   1   JCK-2
.................................................................................................................................................uuggcgugagagacuugguagU..................................................................................   12   1   LT-3
.................................................................................................................................................uuggcgugagagacuugguagU..................................................................................   12   1   JCK-2
..................................................................................................................................................uggcgugagagacuuggu.....................................................................................   2   0   LT-1
..................................................................................................................................................uggcgugagagacuuggu.....................................................................................   6   0   LT-3
..................................................................................................................................................uggcgugagagGcuuggu.....................................................................................   1   1   LT-3
..................................................................................................................................................uggcgugagagacuuggA.....................................................................................   2   1   JCK-1
..................................................................................................................................................uggcgugagagacuuggu.....................................................................................   7   0   JCK-3
..................................................................................................................................................uggcgugagagacuuggu.....................................................................................   8   0   JCK-1
..................................................................................................................................................uggcgugagagacuugUu.....................................................................................   1   1   JCK-2
..................................................................................................................................................uggcgugagagacuuggu.....................................................................................   6   0   LT-2
..................................................................................................................................................uggcgugagagacuuggu.....................................................................................   10   0   LCK-3
..................................................................................................................................................uggcgugagagacuuggu.....................................................................................   6   0   JCK-2
..................................................................................................................................................uggcgugagagacuuggua....................................................................................   41   0   JCK-1
..................................................................................................................................................uggcgugagagacuuggua....................................................................................   30   0   LT-1
..................................................................................................................................................uggcgugagagacuuggua....................................................................................   41   0   LT-2
..................................................................................................................................................uggcgugagagacuugguC....................................................................................   4   1   LCK-3
..................................................................................................................................................uggcgugagagacuugguU....................................................................................   7   1   LCK-3
..................................................................................................................................................uggcgugagagacuuggua....................................................................................   12   0   JT-1
..................................................................................................................................................uggcgugagagaAuuggua....................................................................................   1   1   LT-2
..................................................................................................................................................uggcgugagagacuuggua....................................................................................   42   0   LCK-3
..................................................................................................................................................uggcgugagagacuuggua....................................................................................   51   0   LT-3
..................................................................................................................................................uggcgugagagacuugguC....................................................................................   1   1   JCK-2
..................................................................................................................................................uggcgugagagacuugguU....................................................................................   6   1   JCK-2
..................................................................................................................................................uggcgugagagacuuggua....................................................................................   9   0   JT-2
..................................................................................................................................................uggcgugagagacuuggua....................................................................................   50   0   JCK-2
..................................................................................................................................................uggcgugagagacuugguU....................................................................................   2   1   JCK-1
..................................................................................................................................................uggcgugagagacuugguU....................................................................................   1   1   LT-3
..................................................................................................................................................uggcgugagagacuuggua....................................................................................   5   0   JT-3
..................................................................................................................................................uggcgugagagacuugguC....................................................................................   2   1   JCK-1
..................................................................................................................................................uggcgugagagacuuggua....................................................................................   47   0   JCK-3
..................................................................................................................................................uggcgugagagacuugguaA...................................................................................   3   1   LT-2
..................................................................................................................................................uggcgugagagaAuugguag...................................................................................   1   1   LT-2
..................................................................................................................................................uggcgugagagacuugguag...................................................................................   26   0   LT-3
..................................................................................................................................................uggcgugagagacuugguag...................................................................................   18   0   JT-2
..................................................................................................................................................uggcgugagagacuugguag...................................................................................   38   0   JCK-2
..................................................................................................................................................uggcAugagagacuugguag...................................................................................   1   1   JCK-1
..................................................................................................................................................uggcgugagagacuugguaU...................................................................................   1   1   LT-1
..................................................................................................................................................uggcgugagagacuugguag...................................................................................   13   0   LT-1
..................................................................................................................................................uggcgugagagacuugguag...................................................................................   25   0   JT-1
..................................................................................................................................................uggcgugagagacuugguag...................................................................................   8   0   LCK-3
..................................................................................................................................................uggcgugagagacuugguag...................................................................................   35   0   JCK-1
..................................................................................................................................................uggcgugagCgacuugguag...................................................................................   1   1   LT-3
..................................................................................................................................................uggcgugagagacuugguag...................................................................................   20   0   LT-2
..................................................................................................................................................uggcgugagagacuugguag...................................................................................   23   0   JT-3
..................................................................................................................................................uggcgugagagacuugguag...................................................................................   29   0   JCK-3
..................................................................................................................................................uggcgugCgagacuugguag...................................................................................   1   1   JCK-3
..................................................................................................................................................uggcgugagagacuugguagC..................................................................................   1   1   JCK-3
..................................................................................................................................................uggcgugagagacuugguaga..................................................................................   54   0   LT-2
..................................................................................................................................................uggcgugagagacuugguagU..................................................................................   1   1   JCK-2
..................................................................................................................................................uggcgugagagCcuugguaga..................................................................................   1   1   JCK-3
..................................................................................................................................................uggcgugagagacuugguagC..................................................................................   1   1   LT-3
..................................................................................................................................................uggcgugagagacuugguaga..................................................................................   25   0   JCK-3
..................................................................................................................................................uggcgugagagacuugguaga..................................................................................   42   0   LT-1
```

Mature Star

ucacgugauaacgcguccuuugcuuuuuauuuuuuauuugaaauucaaaaaaauaauaauaaagauauauuuauauaauuguggaugugaaagaagaacuacuaccacuacacagagugauuuuuuuaugaggcugagaaaauuguuggcgugagagacuuggua gaaaucuggguuuuuguuuggacacagaguuaggacuauucuucuuccgaguccgcccaugccaauaauuuccuccgucuuuuc			
uggcgugagagacuugguaga	29	0	JT-1
uggcgugagagacuugguaga	36	0	LCK-3
uggcgugagagacuugguagU	1	1	LT-3
uggcgugagagacuugguaga	80	0	LT-3
uggcgugagagacuugguaga	6	0	JT-2
uggcgugagagacuugguaga	14	0	JT-3
uggcgugCgagacuugguaga	1	1	LT-1
uggcgugagagacuugguaga	27	0	JCK-2
uggcgugagagacuugguaga	22	0	JCK-1
uggcgugagagacuugguagaa	1	0	JCK-2
uggcgugagagacuugguagaa	2	0	JCK-1
uggcgugagagacuugguagaa	1	0	LT-1
uggcgugagagacuugguagaU	4	1	LT-3
uggcgugagagacuugguagaU	4	1	LT-2
cgugagagacuugguaga	1	0	JCK-3
cgugagagacuugguagaaau	1	0	LT-2
cgugagagacuugguagaaauc	1	0	LT-3
cgugagagacuugguagaaaucu	1	0	LT-1
gugagagacuugguagaaauc	2	0	LT-2
ugagagacuugguagaaa	1	0	LT-1
ugagagacuugguagaaa	3	0	LT-3
ugagagacuugguagaaau	1	0	LT-3
ugagagacuugguagaaaucu	1	0	JCK-2
ugagagacuugguagaaaucuggg	2	0	JCK-2
ugagagacuugguagaaaucugggu	1	0	LT-1
acuugguagaaaucugggu	3	0	LT-3
uuuguuuggacacagaguuaggacuau	1	0	JCK-3
uuuguuuggacacagaguuaggacuau	1	0	JCK-2
uuuggacacagaUuuaggacuauu	1	1	LT-3
uuggacacagaUuuaggacu	1	1	LT-1
uuggacacagaguuaggacu	1	0	JCK-3
uuggacacagaUuuaggacua	2	1	LT-2
uuggacacagaUuuaggacua	1	1	LT-1
uuggacacagaguuaggacuau	3	0	LCK-3
acacagaUuuaggacuauucuu	1	1	LT-3
cacagaUuuaggacuauu	2	1	LT-3
cacagaUuuaggacuauucu	2	1	LT-1
cacagaUuuaggacuauucuu	2	1	JCK-3
cacagaUuuaggacuauucuu	1	1	JCK-1
cacagaUuuaggacuauucuu	1	1	LT-3
cacagaUuuaggacuauucuuc	1	1	JCK-3
cacagaguuaggacuauucuucuucc	1	0	LT-3
acagaUuuaggacuauucuucu	2	1	JCK-1
acagaUuuaggacuauucuucu	1	1	JCK-3
cagaUuuaggacuauucuucu	1	1	JCK-3
cagaguuaggacuauucuucuuccgag	1	0	LT-1
uucuuccgaguccgcccaugcc	1	0	LT-3
ucuuccgaguccgcccaug	1	0	JCK-2
ucuuccgaguccgcccaug	3	0	LCK-3
cuuccgaguccgcccaugc	2	0	LT-2
cuuccgaguccgcccaugc	3	0	LCK-3
cuuccgaguccgcccaugc	1	0	LT-3
cuuccgaguccgcccaugc	1	0	LT-1
cuuccgaguccgcccaugU	2	1	LT-1
cuuccgaguccgcccaugcc	2	0	LT-1
cuuccgaguccgcccaugcc	1	0	LT-3
cuuccgaguccgcccaugcc	5	0	LCK-3
cuuccgaguccgcccaugcc	3	0	LT-2
cuuccgaguccgcccaugcca	1	0	LCK-3
cuuccgaguccgcccaugccaaua	5	0	JT-2
cuuccgaguccgcccaugccaaua	3	0	JT-3
uuccgaguccgcccaugc	11	0	LCK-1
uuccgaguccgcccaugc	44	0	LT-2
uuccgaguccgcccaugU	1	1	LT-1
uuccgaguccgcccaugc	97	0	LCK-3
uuccgaguccgcccaugc	3	0	JCK-3
uuccgaguccgcccaugc	5	0	LCK-2
uuccgaguccgcccaugc	8	0	JCK-1
uuccgaguccgcccaugc	2	0	JT-2
uuccgaguccgcccaugc	10	0	JCK-2

Mature Star

```
ucacgugauaacgcguccuuugcuuuuuauuuuuuauuugaaauucaaaaaaauaauaauaaagauauauuuauauaauuguggaugugaaagaagaacuacuaccacuacacagagugauuuuuuuaugaggcugagaaaauguuggcgugagagacuugguagaaaucuggguuuuuguuuggacacagaguuaggacuauucuucuuccgaguccgcccaugccaauaauuuccuccgucuuuuuc
.................................................................................................................................................................................................................uuccgaguccgcccaugc.......................   51    0   LT-1
.................................................................................................................................................................................................................uuccgaguccgcccaugc.......................   12    0   JT-1
.................................................................................................................................................................................................................uuccgaguccgcccaugc.......................   88    0   LT-3
.................................................................................................................................................................................................................uuccgaguccgcccaugc.......................   5     0   JT-3
.................................................................................................................................................................................................................uuccgaguccgcccaugcc......................   9     0   JT-1
.................................................................................................................................................................................................................uuccgaguccgcccaugcU......................   8     1   LT-1
.................................................................................................................................................................................................................uuccgaguccgcccaugcc......................   10    0   JT-2
.................................................................................................................................................................................................................uuccgaguccgcccaugcU......................   6     1   LT-3
.................................................................................................................................................................................................................uuccgaguccgcccaugcU......................   2     1   JCK-1
.................................................................................................................................................................................................................uuccgaguccgcccaugcc......................   29    0   JCK-1
.................................................................................................................................................................................................................uNccgaguccgcccaugcc......................   1     1   JCK-1
.................................................................................................................................................................................................................uuccgaguccgcccaugcc......................   186   0   LT-3
.................................................................................................................................................................................................................uuccgaguccgcccaugcU......................   16    1   LT-2
.................................................................................................................................................................................................................uuccgaguccgcccaugcc......................   160   0   LCK-3
.................................................................................................................................................................................................................uuccgaguccgcccaugcc......................   41    0   JCK-2
.................................................................................................................................................................................................................uuccgaguccgcccaugcU......................   1     1   JCK-2
.................................................................................................................................................................................................................uuccgaguccgcccaugcA......................   1     1   LT-2
.................................................................................................................................................................................................................uuccgaguccgcccaugcc......................   36    0   JCK-3
.................................................................................................................................................................................................................uuccgCguccgcccaugcc......................   1     1   LCK-3
.................................................................................................................................................................................................................uuccgaguccgcccaugcc......................   108   0   LT-2
.................................................................................................................................................................................................................uuccgaguccgcccaugcU......................   10    1   LCK-3
.................................................................................................................................................................................................................uuccgaguccgcccaugcc......................   119   0   LT-1
.................................................................................................................................................................................................................uuccgaguccgcccaugcc......................   14    0   JT-3
.................................................................................................................................................................................................................uuccgaguccgcccaugccU.....................   1     1   LT-3
.................................................................................................................................................................................................................uuccgaguccgcccaugcca.....................   37    0   JT-1
.................................................................................................................................................................................................................uuccgaguccgcccaugcca.....................   28    0   JCK-2
.................................................................................................................................................................................................................uuccgaguccgcccaugcca.....................   16    0   JCK-1
.................................................................................................................................................................................................................uuccgaguccgcccaugccC.....................   1     1   JCK-2
.................................................................................................................................................................................................................uuccgaguccgcccaugccU.....................   1     1   LT-2
.................................................................................................................................................................................................................uuccgCguccgcccaugcca.....................   1     1   JT-1
.................................................................................................................................................................................................................uuccgaguccgcccaugcca.....................   33    0   LT-2
.................................................................................................................................................................................................................uuccgaguccgcccCugcca.....................   1     1   LT-2
.................................................................................................................................................................................................................uuccgaguccgcccaugcca.....................   1     0   JT-2
.................................................................................................................................................................................................................uuccgaguccgcccaugcca.....................   19    0   JCK-3
.................................................................................................................................................................................................................uuccgaguccgcccaugcca.....................   40    0   LT-1
.................................................................................................................................................................................................................uuccgaguccgcccaugccC.....................   1     1   LT-2
.................................................................................................................................................................................................................uuccgaguccgcccaugcUa.....................   1     1   LT-1
.................................................................................................................................................................................................................uuccgaguccgcccaugcca.....................   65    0   LT-3
.................................................................................................................................................................................................................uuccgaguccgcccaugcca.....................   7     0   JT-3
.................................................................................................................................................................................................................uuccgaguccgcccaugcca.....................   59    0   LCK-3
.................................................................................................................................................................................................................uuccgaguccgcccaugccaa....................   3     0   JCK-2
.................................................................................................................................................................................................................uuccgaguccgcccaugccaU....................   1     1   LT-1
.................................................................................................................................................................................................................uuccgaguccgcccaugccaa....................   11    0   LT-1
.................................................................................................................................................................................................................uuccgaguccgcccaugccaa....................   2     0   JCK-1
.................................................................................................................................................................................................................uuccgaguccgcccaugccaa....................   12    0   LT-3
.................................................................................................................................................................................................................uuccgaguccgcccaugccaa....................   13    0   LT-2
.................................................................................................................................................................................................................uuccgaguccgcccaugccaa....................   1     0   JCK-3
.................................................................................................................................................................................................................uuccgaguccgcccaugccaa....................   24    0   LCK-3
.................................................................................................................................................................................................................uuccgaguccgcccaugccaa....................   19    0   LCK-2
.................................................................................................................................................................................................................uuccgaguccgcccaugccaa....................   16    0   LCK-1
.................................................................................................................................................................................................................uuccgaguccgcccaugccaau...................   19    0   LT-2
.................................................................................................................................................................................................................uuccgaguccgcccaugccUau...................   2     1   JCK-2
.................................................................................................................................................................................................................uuccgaguccgcccaugccaau...................   26    0   LCK-1
.................................................................................................................................................................................................................uuccgaguccgcccaugccaau...................   115   0   JCK-2
.................................................................................................................................................................................................................uuccgaguccgcccaugccaau...................   91    0   JCK-3
.................................................................................................................................................................................................................uuccgaguccgcccaugccaau...................   76    0   JCK-1
.................................................................................................................................................................................................................uuccgCguccgcccaugccaau...................   1     1   JCK-3
.................................................................................................................................................................................................................uuccgaguccgcccaugccaau...................   14    0   LCK-2
.................................................................................................................................................................................................................uuccgCguccgcccaugccaau...................   2     1   JCK-2
.................................................................................................................................................................................................................uuccgaguccgcccaugccaau...................   10    0   LCK-3
.................................................................................................................................................................................................................uuccgaguccgcccaugccaCu...................   1     1   JCK-2
.................................................................................................................................................................................................................uuccgaguccgcccaugcAaau...................   1     1   JCK-3
.................................................................................................................................................................................................................uuccgaguccgcccaugccaau...................   48    0   LT-1
.................................................................................................................................................................................................................uuccgaguccgcccaugccaau...................   62    0   LT-3
.................................................................................................................................................................................................................uuccgaguccgcccaugccUau...................   1     1   JCK-1
.................................................................................................................................................................................................................uucUgaguccgcccaugccaau...................   1     1   JCK-3
.................................................................................................................................................................................................................uuccgaguccgcccaugccaauU..................   8     1   LT-2
.................................................................................................................................................................................................................uuccgaguccgcccaugccaauU..................   17    1   LT-3
.................................................................................................................................................................................................................uuccgaguccgcccaugccaauU..................   16    1   JCK-1
.................................................................................................................................................................................................................uuccgaguccgcccaugccaauU..................   4     1   LCK-3
```

```
                                                                                                                                                       Mature                                                          Star
ucacgugauaacgcguccuuugcuuuuuauuuuuuauuugaaauucaaaaaaauaauaauaaagauauauuuauauaauuguggaugugaaagaagaacuacuaccacuacacagagugauuuuuuuaugaggcugagaaaauuguuggcgugagagacuugguagaaaucuggguuuuuguuuggacacagaguuaggacuauucuucuuccgaguccgcccaugccaauaauuuccuccgucuuuuc
.................................................................................................................................................................................................................uuccgaguccgcccaugccaauU.................   24   1   JCK-2
.................................................................................................................................................................................................................uuccgaguccgcccaugccaauU.................   23   1   LT-1
.................................................................................................................................................................................................................uuccgaguccgcccaugccaaua.................   1    0   LT-2
.................................................................................................................................................................................................................uuccgaguccgcccaugccaauU.................   27   1   JCK-3
.................................................................................................................................................................................................................uuccgaguccgcccaugccaauUa................   3    1   JCK-3
.................................................................................................................................................................................................................uuccgaguccgcccaugccaauUa................   1    1   LT-3
.................................................................................................................................................................................................................uuccgaguccgcccaugccaauUa................   4    1   JCK-2
.................................................................................................................................................................................................................uuccgaguccgcccaugccaauUa................   2    1   JCK-1
.................................................................................................................................................................................................................uuccgaguccgcccaugccaauUau...............   1    1   LT-1
.................................................................................................................................................................................................................uuccgaguccgcccaugccaauaUu...............   1    1   LT-1
..................................................................................................................................................................................................................uccgaguccgcccaugcc.....................   1    0   JCK-3
..................................................................................................................................................................................................................uccgaguccgcccaugcc.....................   1    0   LT-1
..................................................................................................................................................................................................................uccgaguccgcccaugcca....................   1    0   JCK-3
..................................................................................................................................................................................................................uccgaguccgcccaugcca....................   1    0   LT-1
..................................................................................................................................................................................................................uccgaguccgcccaugccaa...................   2    0   LT-2
..................................................................................................................................................................................................................uccgaguccgcccaugccaau..................   2    0   JCK-2
..................................................................................................................................................................................................................uccgaguccgcccaugccaau..................   1    0   LT-2
..................................................................................................................................................................................................................uccgaguccgcccaugccaau..................   2    0   JCK-3
..................................................................................................................................................................................................................uccgaguccgcccaugccaaua.................   4    0   JCK-1
..................................................................................................................................................................................................................uccgaguccgcccaugccaaua.................   2    0   JCK-3
..................................................................................................................................................................................................................uccgaguccgcccaugccaauU.................   1    1   JCK-2
..................................................................................................................................................................................................................uccgaguccgcccaugccaaua.................   5    0   JCK-2
...................................................................................................................................................................................................................ccgaguccgcccaugccaaua.................   2    0   JCK-3
...................................................................................................................................................................................................................ccgaguccgcccaugccaaua.................   1    0   JCK-2
..................................................................................................................................................................................................................................ccaauaauuuccuccgucu....   1    0   LT-3
```
